# Associations Between Prenatal Exposure to Serotonergic Medications and Biobehavioral Stress Regulation: Protocol for a Systematic Review and Meta-analysis

**DOI:** 10.2196/33363

**Published:** 2022-03-28

**Authors:** Enav Z Zusman, Alekhya Lavu, Colleen Pawliuk, Jodi Pawluski, Sarah M Hutchison, Robert W Platt, Tim F Oberlander

**Affiliations:** 1 Department of Pediatrics Faculty of Medicine University of British Columbia Vancouver, BC Canada; 2 Department of Obstetrics & Gynaecology University of British Columbia Vancouver, BC Canada; 3 British Columbia Children’s Hospital Research Institute Vancouver, BC Canada; 4 College of Pharmacy University of Manitoba Winnipeg, MB Canada; 5 Univ Rennes, Inserm, EHESP Irset (Institut de Recherche en Santé, Environnement et Travail) UMR_S 1085, F-35000 Rennes France; 6 Centre for Clinical Epidemiology Lady Davis Institute Jewish General Hospital Montreal, QC Canada; 7 Department of Pediatrics Faculty of Medicine and Health Sciences McGill University Montreal, QC Canada; 8 Department of Epidemiology, Biostatistics, and Occupational Health Faculty of Medicine and Health Sciences McGill University Montreal, QC Canada; 9 School of Population and Public Health University of British Columbia Vancouver, BC Canada

**Keywords:** pregnancy, serotonergic medications, antidepressants, stress regulation, systematic review, meta-analysis

## Abstract

**Background:**

Up to 20% of mothers experience antenatal depression and approximately 30% of these women are treated with serotonergic psychotropic pharmacological therapy during pregnancy. Serotonergic antidepressants readily cross the placenta and the fetal blood-brain barrier, altering central synaptic serotonin signaling and potentially altering serotonin levels in the developing fetal brain.

**Objective:**

The aim of this study is to assess the impact of prenatal exposure to serotonergic antidepressants, accounting for maternal mood disturbances, on markers of stress regulation during childhood.

**Methods:**

We will follow PRISMA (Preferred Reporting Items for Systematic Reviews and Meta-Analyses) guidelines and will search MEDLINE, Embase, CINAHL, PsycINFO, and ClinicalTrials.gov for full-length studies that assessed physiological (eg, cortisol level, heart rate variability, salivary amylase, pupillary size, C-reactive protein) indices of stress regulation in children of pregnant people who were treated with a serotonergic antidepressant at any point during pregnancy. We will assess the quality of observational studies using the Newcastle-Ottawa Scale and the quality of experimental studies using the Cochrane risk-of-bias tool. When possible, we will conduct a random-effects meta-analysis. If meta-analysis is not possible, we will conduct a narrative review. If a sufficient number of studies are found, we will perform subgroup analysis and assess outcomes measured by drug class, dose, trimester of exposure, and child’s age and gender.

**Results:**

We registered our review protocol with PROSPERO (International Prospective Register of Systematic Reviews; CRD42021275750), completed the literature search, and initiated title and abstract review in August 2021. We expect to finalize this review by April 2022.

**Conclusions:**

Findings should identify the impact of prenatal antidepressant effects on stress regulation and distinguish it from the impact of prenatal exposure to maternal mood disturbances. This review should inform decisions about serotonergic antidepressant use during pregnancy.

**Trial Registration:**

PROSPERO CRD42021275750; https://www.crd.york.ac.uk/prospero/display_record.php?RecordID=275750

**International Registered Report Identifier (IRRID):**

PRR1-10.2196/33363

## Introduction

### Background

Up to 20% of mothers experience antenatal depression and approximately 30% of these women are treated with a serotonergic antidepressant during pregnancy [1,2]. Selective serotonin reuptake inhibitors (SSRI) are the most common serotonergic medications prescribed [3-7]. They readily cross the placenta and the fetal blood-brain barrier, potentially altering serotonin (or 5-hydroxytryptamine [5-HT]) signaling in the fetal brain [8-14], and such exposure has been paradoxically reported to be associated with an increased risk for anxiety, attention, and behavioral disorders in children of mothers with depression treated with an SSRI during pregnancy [15-17]. Importantly, childhood behaviors have been associated with altered indices of stress regulation [18-21], raising critical questions about whether prenatal exposure to serotonergic psychotropic medications alters stress reactivity, thereby contributing to an increased risk for behavioral disturbances.

Long before 5-HT becomes a neurotransmitter in the mature brain, it plays a role as a neurodevelopmental signal regulating cell growth and function [22,23]. In the fetal brain, 5-HT and its receptors are overexpressed and widespread in regions where they are absent in adults, pointing to a time-dependent specificity to 5-HT expression during development [24,25]. Early 5-HT alterations, either via pharmacological, genetic, or other manipulations, potentially alter these processes via the presynaptic, membrane-bound serotonin transporter protein (5-HTT), the target of SRI antidepressants. 5-HTT is a key regulator of brain 5-HT [26,27].

Serotonin is central to the development and function of two key stress response systems—the locus-coeruleus-norepinephrine (autonomic nervous system [ANS]) and the hypothalamic-pituitary-adrenal (HPA) systems [28-30], which may illustrate sites affected by prenatal exposure to serotonin reuptake inhibitors on stress responses [31-33]. The relationship between 5-HT and stress reactivity is bidirectional; stressors appear to alter 5-HT metabolism as well as bias how one copes with subsequent stressful challenges [32,34]. ANS activation leads to a rapid “flight or fight” response, in turn leading to increased cardiac activity (heart rate) and release of catecholamines (norepinephrine) [35]. Central to our understanding of how prenatal exposure to serotonergic medications such as antidepressants influences early brain development is understanding the diverse roles the neurotransmitter 5-HT plays in early brain development, stress regulation, and mental health [23,28,36]. Prenatal maternal mood disturbances, the very disorders that lead to antidepressant treatment, have also been shown to shape the development of the HPA axis [37,38].

5-HT and cardiovascular/autonomic stress regulation are highly interrelated via links between reflex control of parasympathetic outflow to the heart and other organs that involve central 5-HT1A receptors located in the vicinity of preganglionic vagal neurons. Further, 5-HT3 receptors are implicated in afferent regulation of central sympathetic and parasympathetic tone [39]. The development and function of the HPA stress response and the serotonergic regulatory systems are highly interrelated and exquisitely sensitive to the effects of early adverse experience [40,41]. Serotonin influences how an individual copes with subsequent social stressors and plays a role in mediating the effects of adverse experience [42]. Early differences in maternal care alter central 5-HT levels that change HPA axis stress function, reflected as an altered capacity to regulate stress responses [40,43].

Considering the importance of serotonin in neurodevelopment, it is conceivable that early changes to 5-HT, secondary to prenatal serotonergic medication exposure or maternal mood disorders, could have developmental consequences [26,44] and may modify the formation and function of key stress regulatory systems such as the ANS and HPA axis in ways that may affect subsequent responses to stress challenges and may have life-long implications for the offspring’s health and behavior. Understanding relationships between stress response systems and children’s behavior may provide essential insight into the developmental origins of physiological processes that contribute to disrupted behavior. Taken together, it is possible that prenatal exposure to serotonergic psychotropic medications used to manage mood disturbances during pregnancy could alter stress reactivity/regulation in offspring.

### Objectives

Our study aims to assess the impact of prenatal exposure to serotonergic medications, and distinguish these effects from the impact of maternal mood disturbances on neonatal, infant, childhood, and adolescent indices of stress regulation.

## Methods

### Overview

We will adhere to the PRISMA (Preferred Reporting Items for Systematic Reviews and Meta-Analyses) guidelines for reporting systematic reviews [45] and have used the PRISMA for systematic review protocols (PRISMA-P) [46]. Our research protocol was designed a priori, defining methods for searching the literature, including and examining articles, and extracting and analyzing data.

### Eligibility Criteria

#### Inclusion Criteria

This systematic review will consider studies that included pregnant people diagnosed with prenatal mood disorders (depression and/or anxiety) who were exposed to serotonergic medications at any point during pregnancy. We will assess stress regulation outcomes in the offspring and the way they relate to behavior. We will include monopharmacy use of antidepressants. We will include both singleton and multiple gestation pregnancies as well as both nulliparous and multiparous** **pregnancies. We will include intervention studies (randomized controlled trials, pre-post trials) and observational trials (case-control studies, cross-sectional studies, cohort studies, case reports or case series). We will only include full-text studies published in English or French. Studies that meet our inclusion criteria will be included in this review.

#### Exclusion Criteria

We will exclude polypharmacy use of multiple antidepressant medications from several classes, as well as animal studies, gray literature (including theses and dissertations), review studies, letters to the editor, conference abstracts, and posters. If we come across different studies that include the same population and outcome, the study that involves a longer follow-up will be included.

### Outcome Measures

We will assess physiological outcomes and how they relate to each other. Specifically, our outcome includes the following physiological outcomes: cortisol, heart rate variability, salivary amylase, pupillary size, C-reactive protein (CRP), and immunological biomarkers (cytokines, chemokines, lymphokines, IL-6, etc).

### Information Sources and Literature Search

We developed our search strategy with the consultation of a librarian and will search the literature by population and intervention (Table 1). The following databases will be searched: MEDLINE (Ovid), Embase (Ovid), CINAHL (EBSCOhost), PsycINFO (EBSCOhost), and ClinicalTrials.gov. We will use both Medical Subject Headings (MeSH) terms and keywords. Our search strategy for the MEDLINE database is outlined in Multimedia Appendix 1. We will adjust the MeSH terms and keywords used to accommodate the different databases’ requirements and limitations. We will limit our search to studies published in English or French. We will not limit for year of publication and will include original studies of all study types.

**Table 1:** Eligibility criteria to be included in the review.

Item	Criteria
Population	Pregnant people diagnosed with antepartum depression, prenatal depression, or maternal mood disorders
Intervention/exposure	Exposure to a serotonergic drug: SSRI^a^: citalopram, escitalopram, fluoxetine, fluvoxamine, paroxetine, sertraline SNRI^b^: desvenlafaxine, duloxetine, levomilnacipran, venlafaxine Second-generation antipsychotics: aripiprazole, brexpiprazole, olanzapine, quetiapine, risperidone Serotonin modulators: trazodone, vilazodone, vortioxetine Tricyclic antidepressants: amitriptyline, clomipramine, desipramine, doxepin, imipramine, nortriptyline, trimipramine 5-HT1A (serotonin receptor) agonist: buspirone
Comparison/control	None
Outcome	Physiological outcomes: cortisol, cardiac autonomic function (heart rate variability, pre-ejection period), salivary amylase, pupillary size, C-reactive protein, immunological biomarkers (cytokines, chemokines, lymphokines, IL-6).

^a^SSRI: selective serotonin reuptake inhibitor.

^b^SNRI: serotonin–norepinephrine reuptake inhibitor.

We will use the following keywords and MeSH terms: Pregnancy Trimesters/ or Pregnancy/ or Pregnancy Trimester, Third/ or pregnancy.mp. or Pregnancy Trimester, First/ or Pregnancy, or Pregnancy Trimester, Second/, pregnant.mp. or Pregnant Women/, or gestation.mp. or perinatal.mp. or prenatal.mp. or pregnant* AND Depression/ or depressive disorder/ or depressive disorder, major/ or depressive disorder, treatment-resistant/ or Anxiety Disorders/ or Anxiety/ or anxiety.mp., or Mood Disorders/, AND antidepressants.mp. or Antidepressive Agents/, serotonergic drugs.mp. or Serotonin Agents/, selective serotonin reuptake inhibitors.mp. or Serotonin Uptake Inhibitors/, citalopram.mp. or Citalopram/, escitalopram.mp. or Citalopram/, fluoxetine.mp. or Fluoxetine/, fluvoxamine.mp. or Fluvoxamine/, paroxetine.mp. or Paroxetine/, sertraline.mp. or Sertraline/, “Serotonin and Noradrenaline Reuptake Inhibitors”/ or Serotonin Uptake Inhibitors/ or serotonin-norepinephrine reuptake Inhibitor.mp., desvenlafaxine.mp., or Desvenlafaxine Succinate/, duloxetine.mp. or Duloxetine Hydrochloride/, levomilnacipran.mp. or Levomilnacipran/, venlafaxine.mp. or Venlafaxine Hydrochloride/, Antipsychotic Agents/ or Second generation antipsychotics.mp., aripiprazole.mp., or Aripiprazole/, brexpiprazole.mp., olanzapine.mp., or Olanzapine/, quetiapine.mp. or Quetiapine Fumarate, risperidone.mp. or Risperidone/, Serotonin Modulators.mp., trazodone.mp., or Trazodone/, vilazodone.mp. or Vilazodone Hydrochloride/, vortioxetine.mp. or Vortioxetine/, tricyclic antidepressants.mp. or Antidepressive Agents, Tricyclic/, amitriptyline.mp. or Amitriptyline/, clomipramine.mp. or Clomipramine/, desipramine.mp. or Desipramine/, doxepin.mp. or Doxepin/, imipramine.mp. or Imipramine/, nortriptyline.mp. or Nortriptyline/, trimipramine.mp. or Trimipramine/ or buspirone.mp. or Buspirone/.

We will conduct a manual search of the journals *Psychoneuroendocrinology*, *Early Human Development*, *Neuroscience*, and *Neuroscience & Biobehavioral Reviews*, as well as a forward and backward citation search through Google Scholar [47] on all included papers to locate additional papers that may have been missed in our literature search.

### Study Selection Process

Using our predetermined selection criteria, two authors (EZZ and AL) will independently screen all retrieved papers at level 1 (title and abstract) for inclusion in the study using Covidence, a screening and data extraction tool for systematic reviews [48]. Once a list of studies is determined, the selected papers will be reviewed at level 2 (full text) to select a final list of review studies. Screening questions can be found in Multimedia Appendix 2. At any point, authors will meet to discuss discrepancies and a third author (SH) will be consulted if disagreement occurs.

### Data Collection Process

Two authors (EZZ and AL) will independently extract the following information from all included studies: year of publication, country, sample size, study design, study setting, trimester of pregnancy, drug exposure class, drug exposure generic name, drug dose, cortisol level (diurnal), cortisol level (stress challenge), heart rate variability, salivary amylase, pupillary size, CRP, cytokines, chemokines, lymphokines, IL-6, maternal depression, maternal depression diagnosis method, maternal anxiety, and maternal anxiety diagnosis method. Authors will meet to discuss discrepancies and a third author (SH) will be consulted if disagreement occurs. If needed, we will contact study corresponding authors for unpublished or missing data.

### Quality and Risk of Bias Assessment

To assess the methodological quality of the included studies and their risk of bias, we will use different checklists and scales. Two authors (EZZ and AL) will independently screen each included paper according to its methodology. We will use the Newcastle-Ottawa Scale for observational cohort studies [49], the modified Newcastle-Ottawa Scale for observational cross-sectional studies [50], and the Cochrane risk-of-bias tool for randomized controlled trials and experimental studies [51]. We will use funnel plots to assess for publication bias [52]. In case of a publication bias, we will use the trim-and-fill method. We will remove (“trim”) the studies that give rise to the funnel plot’s asymmetry and then impute (“fill”) the suggested missing studies based on the bias-corrected overall estimate [53].

### Synthesis of Included Studies

We will pool studies based on their reported outcome and will present the characteristics of included studies both descriptively and in a table. Where possible, we will pool reported levels of cortisol, heart rate variability, salivary amylase, pupillary size, and CRP. We will calculate the Cochrane Q test (chi-square) and Higgins *I*^2^ score to assess the statistical heterogeneity of effect size estimates across our included studies before running a meta-analysis. When meta-analysis is possible, we will calculate pooled mean differences for continuous data and perform a random-effects meta-analysis for dichotomous data [54]. If meta-analysis is not possible, we will conduct a narrative synthesis of the data. When a sufficient number of studies are found, we will perform subgroup analysis and assess outcomes measured by drug class, dose, trimester of exposure, and child’s age and gender. We will consider method of assessment for pooling. We will perform all data analysis using Stata (version 15; StataCorp LLC).

## Results

We registered our review protocol with PROSPERO (International Prospective Register of Systematic Reviews; CRD42021275750) [55], completed the literature search, and initiated title and abstract review in August 2021. We expect to finalize this review by April 2022.

## Discussion

Antepartum depression is a common condition that is often treated with different serotonergic drugs, leading to altered central serotonin signaling in the developing brain. As serotonin plays a key role in fetal neurodevelopment that shapes key components of stress regulation pathways, understanding how prenatal exposure to these medications affects physiological stress responses could elucidate pathways to behavioral outcomes in the offspring of mothers with depression. This study will add to the existing body of knowledge by integrating data that will lead to new insights about early origins of mental health disorders and the risks and benefits of use of serotonergic medications. The impact of prenatal serotonergic medication exposure and early origins of mental health will be essential for both theoretical and clinical reasons, specifically to inform decisions about serotonergic medication use in pregnancy and to inform interventions that promote healthy child development.

## Appendix

Multimedia Appendix 1 Search strategy for MEDLINE database.

Multimedia Appendix 2 Reviewers' literature search screening questions.

## Abbreviations

5-HT: 5-hydroxytryptamine (serotonin)
5-HTT: serotonin transporter
ANS: autonomic nervous system
CRP: C-reactive protein
HPA: hypothalamic-pituitary-adrenal
MeSH: Medical Subject Headings
PRISMA: Preferred Reporting Items for Systematic Reviews and Meta-Analyses
PRISMA-P: Preferred Reporting Items for Systematic Reviews and Meta-Analyses Protocols
PROSPERO: International Prospective Register of Systematic Reviews
SNRI: serotonin–norepinephrine reuptake inhibitor
SSRI: selective serotonin reuptake inhibitor
