# Supplementary figures and images for: Associations Between Prenatal Exposure to Serotonergic Medications and Biobehavioral Stress Regulation: Protocol for a Systematic Review and Meta-analysis
Source: JMIR Res Protoc. 2022 Mar 28;11(3):e33363. doi: 10.2196/33363 (PMC9002587; doi:10.2196/33363)

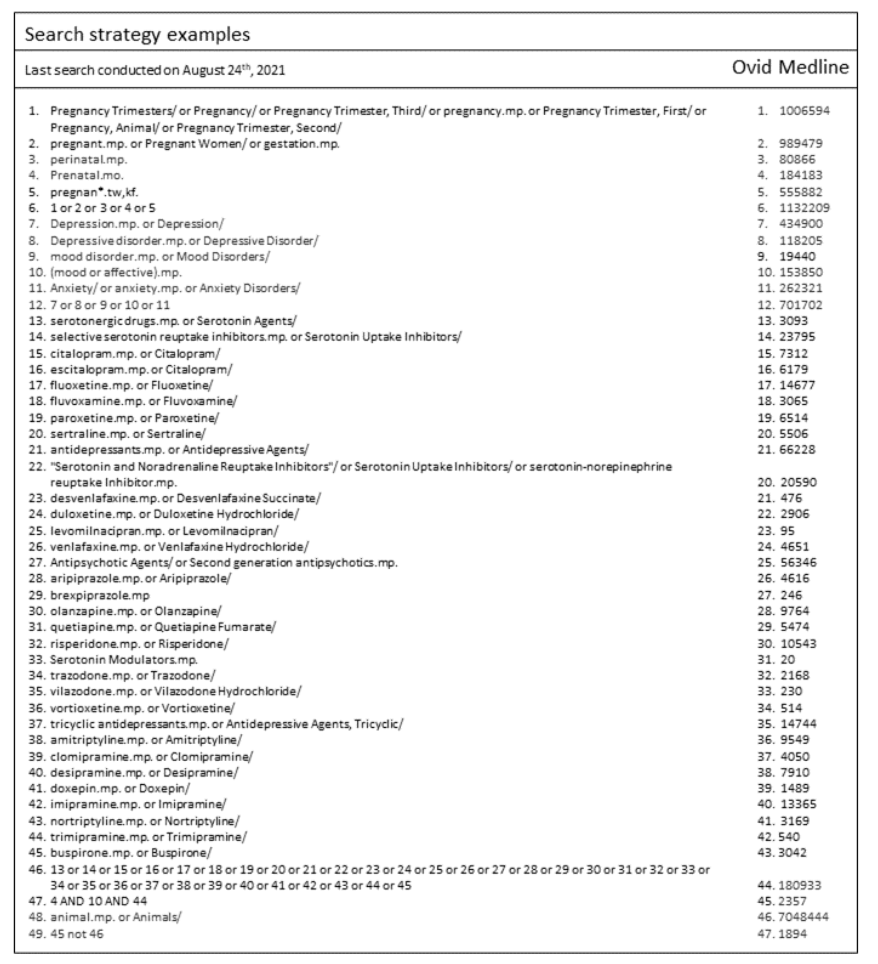

Supplement: Multimedia Appendix 1 [file resprot_v11i3e33363_app1.png]
