# Supplementary material for: Associations Between Prenatal Exposure to Serotonergic Medications and Biobehavioral Stress Regulation: Protocol for a Systematic Review and Meta-analysis
Source: JMIR Res Protoc. 2022 Mar 28;11(3):e33363. doi: 10.2196/33363 (PMC9002587; doi:10.2196/33363)
Supplement: Multimedia Appendix 2 [file resprot_v11i3e33363_app2.docx]

**Multimedia Appendix 2**

**Screening questions for reviewers:**

**Level 1 Screening: Title and Abstract**

1. Was the study conducted exclusively in humans?

YES____ NO____ MAYBE____

1. Is this a relevant study design (Randomized controlled trials or observational studies)?

YES____ NO____ MAYBE____

1. Does the study include pregnant people diagnosed with prenatal mood disorders (depression and/or anxiety)?

YES____ NO____ MAYBE____

1. Does the study include exposure to serotonergic medications at any point during pregnancy?

YES____ NO____ MAYBE____

1. Does the study report at least one outcome of interest related to stress regulation (cortisol, heart rate variability, salivary amylase, pupillary size, C-reactive protein (CRP), immunological biomarkers (cytokines, chemokines, lymphokines, IL-6, etc.)?

YES____ NO____ MAYBE____

Note: All studies that answered YES and MAYBE were included, and NO were excluded.

**Level 2 Screening (Full text)**

1. Is this a relevant study design (Randomized controlled trials or observational studies)?

YES____ NO____

1. Does the study include pregnant people diagnosed with prenatal mood disorders (depression and/or anxiety) who are exposed to serotonergic medications at any point during pregnancy?

YES____ NO____

1. Does the study include pregnant people diagnosed with prenatal mood disorders (depression and/or anxiety) who are exposed to serotonergic medications at any point during pregnancy?

YES____ NO____

1. Does the study report at least one outcome of interest related to stress regulation (cortisol, heart rate variability, salivary amylase, pupillary size, C-reactive protein (CRP), immunological biomarkers (cytokines, chemokines, lymphokines, IL-6, etc.)?

YES____ NO____

Note: All studies that answered YES were included, and NO were excluded.
